# Structure and Dynamics of GPCRs in Lipid Membranes: Physical Principles and Experimental Approaches

**DOI:** 10.3390/molecules25204729

**Published:** 2020-10-15

**Authors:** Andrew J. Y. Jones, Florian Gabriel, Aditi Tandale, Daniel Nietlispach

**Affiliations:** Department of Biochemistry, University of Cambridge, 80 Tennis Court Road, Cambridge CB2 1GA, UK; ajyj2@cam.ac.uk (A.J.Y.J.); fg382@cam.ac.uk (F.G.); ayt22@cam.ac.uk (A.T.)

**Keywords:** GPCR, lipids, membrane mimetics, NMR

## Abstract

Over the past decade, the vast amount of information generated through structural and biophysical studies of GPCRs has provided unprecedented mechanistic insight into the complex signalling behaviour of these receptors. With this recent information surge, it has also become increasingly apparent that in order to reproduce the various effects that lipids and membranes exert on the biological function for these allosteric receptors, in vitro studies of GPCRs need to be conducted under conditions that adequately approximate the native lipid bilayer environment. In the first part of this review, we assess some of the more general effects that a membrane environment exerts on lipid bilayer-embedded proteins such as GPCRs. This is then followed by the consideration of more specific effects, including stoichiometric interactions with specific lipid subtypes. In the final section, we survey a range of different membrane mimetics that are currently used for in vitro studies, with a focus on NMR applications.

## 1. Introduction

**Figure 1:**
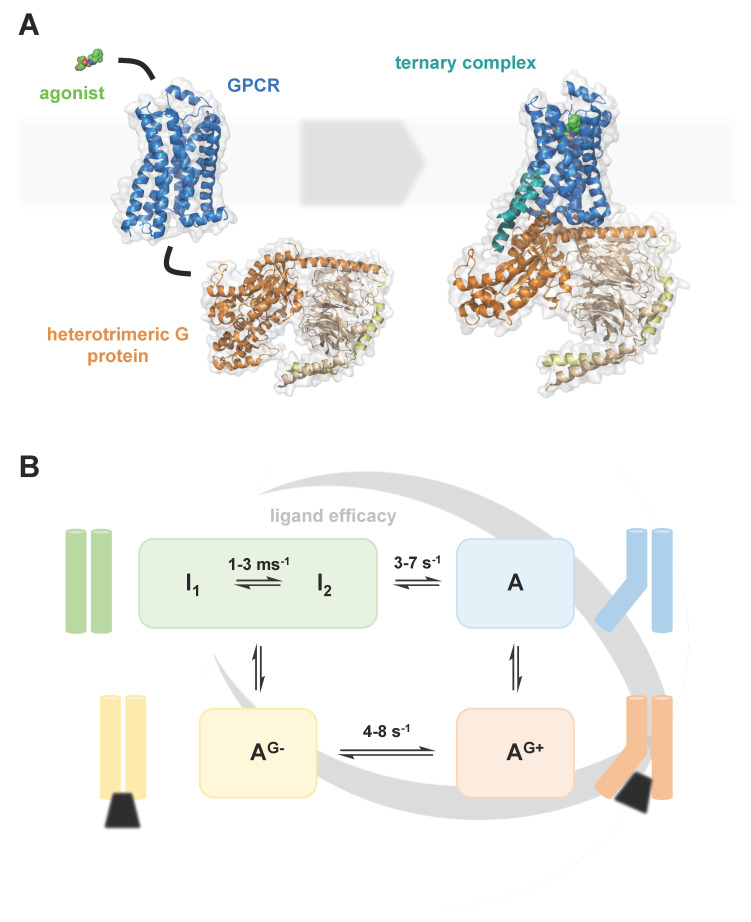
(**A**) Structural basis of GPCR activation. The binding of an agonist (green) induces conformational changes in the GPCR (blue), which allows the latter to interact with cytoplasmic coupling partners (CCPs), such as the heterotrimeric G protein depicted (orange/beige/yellow). In the resulting ternary complex, TM6 and TM5 (cyan) have moved out from the receptor helix bundle. This opens a cytoplasmic cavity in the receptor, which accommodates the CCP. The figure was designed with the crystal structures of the β_2_ adrenergic receptor (β_2_AR) in inactive form bound to antagonist, and in active, agonist-bound ternary state (PDB accession codes: 3NYA, 3SN6, 6EG8). (**B**) Model of the conformational equilibrium of a GPCR based on ^19^F NMR studies of the β_1_ adrenergic receptor (β_1_AR)). Under physiological conditions, GPCRs adapt a complex ensemble of conformational states that are in exchange with each other. I_1_ and I_2_ in this model are two inactive states of the β_1_AR, that exchange at high frequencies on a ms–μs time scale. The pre-active receptor state A is marked by an opening of the intracellular cavity of the β_1_AR. A^G−^ reflects a pre-coupled state of the β_1_AR, in which a CCP binds to an inactive conformation of the receptor. A^G+^ represents the fully active state of the GPCR, in which the CCP binds into the cytoplasmic cavity of the receptor that is opened by the outward movement of TM6. The binding of a ligand or CCP can shift this conformational equilibrium and can thus guide GPCR activation by conformational selection. Ligand efficacy was in this context, e.g., found to correlate well with increased exchange kinetics and the population of more active states [6,7].

G protein-coupled receptors (GPCRs) are 7-transmembrane helix receptors, which are present in all eukaryotic organisms and have homologues in bacteria and archaea. With approximately 800 individual members, GPCRs form one of the largest protein families encoded in the human genome [1]. GPCRs play key roles at virtually all levels of intercellular communication, regulating biological processes from cell growth to synaptic signal transmission. Due to their physiological importance and their good accessibility as cell surface receptors, GPCRs are one of the most important drug targets today and still harbour great pharmacological potential [2]. In recent decades, our understanding of GPCRs has greatly advanced. Especially the determination of by now over 350 GPCR structures by x-ray crystallography and cryo-EM (see, e.g., GPCRdb.org) has led to fascinating insights into the molecular details of these receptors (Figure 1A). The complementation of these structural insights with biophysical techniques, including NMR (e.g., Imai 2020) [3], single-molecule fluorescence (e.g., Lamichhane 2020) [4] and HDX-MS (e.g., Du 2019) [5], provides us today with an expanding mechanistic understanding of GPCR activation and signalling (Figure 1B).

Building on this progress, it is today increasingly appreciated that the membrane environment of GPCRs can significantly influence the structure, dynamics and function of the receptors. As ever more studies shed light on the interactions between GPCRs and the surrounding lipid bilayer, the great complexity of this relationship becomes evident. In this review, we attempt a dissection and organisation of this complexity. We elaborate on both the biological diversity of lipid membranes and the plethora of physical interactions between lipid bilayers and embedded GPCRs. We furthermore provide an overview of the current state and the latest advancements in mimicking lipid membranes for in vitro studies of GPCRs. In this context, we discuss different membrane mimetics in regard to the membrane features they can resemble and their compatibility with different structural and biophysical techniques.

## 2. Cellular Trafficking of Membrane Lipids and GPCRs

A hallmark of eukaryotic cells is their extensive network of intracellular membrane systems. These membrane systems compartmentalise the interior of the cell into the chemically separated and distinct spaces of the cytoplasm, vesicles and different organelles [8]. Apart from differences in the chemical composition of their aqueous phases, these compartments are also marked by a tremendous chemical heterogeneity of their separating lipid membranes [9,10].

In eukaryotic cells, more than 1000 different lipid species come together to form the different lipid membranes [11]. The vast majority of those lipids are synthesised in the membrane of the endoplasmic reticulum (ER), including most glycerophospholipids, ceramides (the precursors of sphingolipids) and sterols [9]. The ER is also the primary site for the incorporation of unsaturated acyl chains into membrane lipids [12]. Newly synthesised lipids are then trafficked through the cell in vesicular membranes.

Most membrane lipids pass at some point the Golgi apparatus. The Golgi is a centre for membrane lipid modification and further trafficking, and in this context is especially important for the synthesis and glycosylation of sphingolipids [9,13,14]. Membrane lipids are eventually trafficked to the plasma and organellar membranes. Even having reached their destined locations, membrane lipids can still be extensively modified or degraded [9]. Consequently, the lipid composition of cellular membranes can vary greatly between distinct membrane systems. The composition can also rapidly change over time, as many lipid species are being chemically modified as an integral part of cellular signalling pathways [15].

The synthesis and trafficking of membrane lipids and GPCRs follow similar routes. GPCRs are conventionally understood in their role as cell surface receptors, residing in the plasma membranes of eukaryotic cells [16,17]. It is, however, important to bear in mind that every GPCR has been a part of various cellular membrane systems over the course of its lifetime. GPCRs are co-translationally inserted into the membrane of the ER [18,19]. They are then trafficked in vesicles along the secretory pathway of the cell, passing the membranes of the ERGIC and the Golgi apparatus [20], before reaching the plasma membrane. Towards the end of their lifetime, GPCRs are internalised from the cell surface into the membranes of endosomes [21]. These can later fuse with lysosomes, where the receptors are eventually degraded [22]. Beyond this “classical” lifecycle of GPCRs as cell surface receptors, an increasing body of studies finds GPCRs being terminally trafficked to organellar membranes [23,24,25]. Several GPCRs have in this context been found to be localised additionally or exclusively to the membranes of, e.g., the nuclear envelope or mitochondria (for an extensive review on organellar GPCRs, see Jong 2018) [26]. The different subcellular localisations of GPCRs imply significant variations in the lipid environments of the receptors, which might play decisive roles in shaping GPCR activation and signalling in a membrane specific manner.

## 3. Interactions of GPCRs with Their Membrane Environment

GPCRs are integral membrane proteins (IMPs) that have a correspondingly intimate relationship with lipid membranes as their immediate physical environment. This relationship is complex in many regards. Beyond the chemical heterogeneity of cellular membranes discussed above, it is the extensive spectrum of interactions between GPCRs and the surrounding membrane that remains difficult to capture. These interactions range from bulk physical properties that a lipid membrane exerts on a GPCR, to specific and stoichiometric interactions between a receptor and individual lipid molecules. In this chapter, we discuss the effects that interactions with both the bulk of a lipid membrane and individual membrane lipids can have on the structure and function of GPCRs.

### 3.1. General Membrane Effects on GPCRs

**Figure 2:**
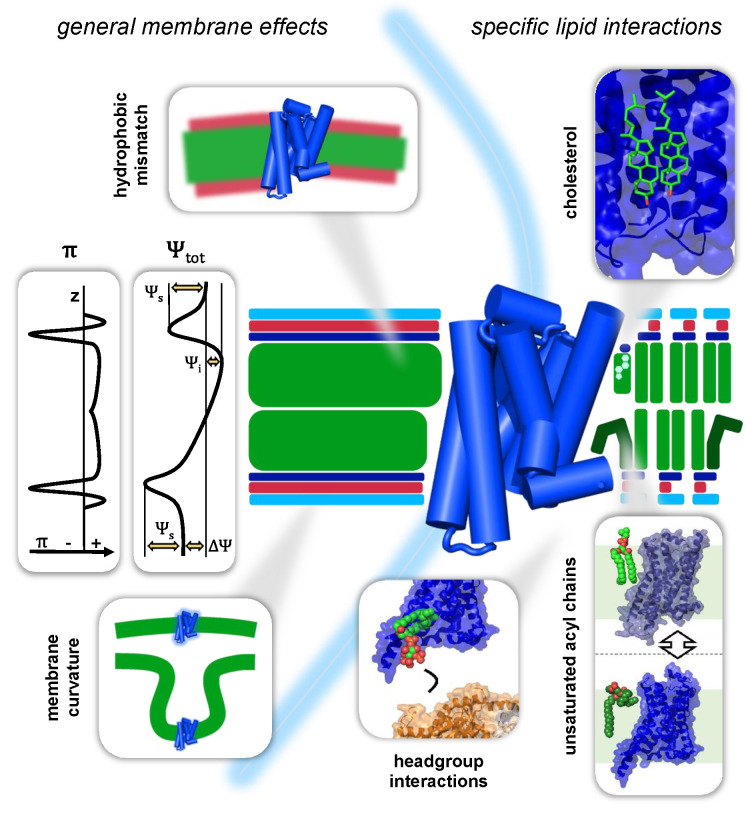
Schematic overview of important interactions of GPCRs with their membrane environment. On the left, general membrane effects are depicted. π: common lateral pressure profile in the z dimension of a lipid bilayer. While the lateral pressure is generally positive in the hydrocarbon core, it is inversed to strong tensions in the carbonyl region. Ψ_tot_: electric potential across a cellular membrane. The graph depicts the sum of the individual potentials ΔΨ (transmembrane potential), Ψ_s_ (surface potential of the lipid headgroups) and Ψ_i_ (potential in the core of the membrane). The cartoon is inspired by Honig (1986) [30]. Membrane curvature: the curvature of membranes can range from near zero to strong membrane-invaginations and highly curved vesicular membranes. Hydrophobic mismatch: the difference in hydrophobic thickness between a lipid bilayer and the TMDs of a GPCR can strongly affect the structure and dynamics of the latter. On the right, examples of specific lipid–GPCR interactions are illustrated. Cholesterol: one important stoichiometric interaction partner of many GPCRs is cholesterol. The figure shows the crystal structure of the human β_2_ adrenergic receptor (blue) in complex with cholesterol molecules (green) (PDB accession code: 2RH1). Headgroup interactions: in the interface region of a membrane, GPCRs interact with lipid headgroups. Several of these interactions have been shown to occur in a lipid-specific and stoichiometric way. The figure shows a model of a PIP2 molecule (green), forming a ternary complex with a GPCR (blue) and a heterotrimeric G protein (orange). The figure was based on findings by Yen et al., 2018 [31] and was designed with crystal structures of the β_2_ adrenergic receptor and a heterotrimeric G protein (PDB accession codes: 3SN6, 6EG8). Unsaturated acyl chains: double bonds in the membrane lipid tails confer distinct physical properties. On the one hand, unsaturated acyl chains can reduce the packing density and other general membrane properties. On the other hand, the distinct chemical properties of unsaturated acyl chains may allow for specific interactions with GPCRs, which could in turn influence the activation of the regarding receptors. Unsaturated acyl chains, as in the lipid POPC (dark green), could, e.g., stabilize more active conformations of a GPCR (PDB accession code: 3SN6), while saturated acyl chains, as in the lipid DPPC, might favour inactive conformations (PDB accession code: 2YCW).

In terms of their spatial dimensions, lipid membranes have a span of approximately 5 nm in the z dimension (orthogonal to the x,y plane or lateral dimension of the membrane; see Figure 2). The hydrophobic hydrocarbon core, formed by the acyl chains of the membrane lipids, occupies approximately 3 nm of the total thickness. It is flanked by respectively approximately 1 nm of interface region, where the polar head groups of the membrane lipids are in contact with the aqueous phases, separated by the lipid membrane (see Figure 2) [27]. This spatial organisation creates a complex physicochemical profile of the GPCR environment and is accompanied by a variety of effects and forces that have direct implications for membrane-embedded GPCRs. The complex profile of lipid membranes is in vivo further complemented by the asymmetry the two lipid leaflets with regard to their chemical composition and geometry. In this review, we will not discuss this asymmetry and its implications in great detail. However, excellent reviews of this topic can be found in, e.g., van Meer, 2011 and Fadeel and Xue, 2015 [28,29].

#### 3.1.1. Mechanical Forces in the Bilayer

A lipid bilayer is in simple terms a 2D array of lipid molecules. This ordered assembly yields a stratification of chemical moieties in the lateral dimension. The forces that result from the homotypic interactions of these moieties, cooperatively build up to considerable pressures and tensions in the x,y plane of the lipid bilayer. This so-called lateral pressure is a general characteristic of every lipid bilayer. In the hydrocarbon core of the bilayer, repulsion between the hydrocarbon lipid tails is the dominating intermolecular force. This repulsion can give rise to lateral pressures up to 500 bar. Towards the interface region, attracting forces between the carbonyls dominate, generating strong tensions between 1000 and 2000 bar. The head group region of the bilayer is then again usually dominated by intermolecular repulsion between negatively charged phosphate moieties [32,33,34]. These interactions give rise to lateral pressure profiles that look for most bilayers comparable to the one depicted in Figure 2 (cf. π). This profile can, however, vary significantly with the composition of the corresponding lipid bilayer. In the hydrocarbon core of the bilayer, unsaturated acyl chains and cholesterol are the main modulators of lateral pressure [32,33,35]. Unsaturated acyl chains occupy more space in the lateral dimension than their saturated counterparts and consequently lead to increased pressures between the acyl chains [36,37]. Cholesterol, in contrast, seems to have more complex but no less pronounced effects on the pressure profile [38]. The lipid head group composition of a bilayer can furthermore decisively influence the lateral pressure in the interface region [37,39]. Cellular membranes are furthermore asymmetric lipid bilayers, which can lead to correspondingly highly asymmetric lateral pressure profiles [40].

IMPs are directly exposed to the lateral pressure of the surrounding lipid bilayer and changes in the pressure profile can have profound effects on their structure and function. For GPCRs, publications on these effects are still comparably rare. However, many studies on other IMPs, including mechanosensitive ion channels [41,42,43] and membrane transporters [44,45], demonstrate that the lateral pressure could have important implications for GPCRs. It is by now established that GPCR activation includes both large scale structural rearrangements, as well as changes in the plasticity of the receptors [46]. It seems therefore tempting to propose that changes in lateral pressure of the surrounding bilayer could differentially stabilise certain conformations of the receptors and thereby either stimulate or impede GPCR activation. These mechanistic considerations have so far been suggested for some mechanosensitive GPCRs [47,48], but might have a much broader implication also for other GPCRs in general.

#### 3.1.2. Membrane Curvature

Curvature is a ubiquitous feature of biological membranes. It ranges in its extent from slight bending as observed in the plasma membrane to the strongly curved membranes of small cellular vesicles. In vivo the curvature of a membrane is often determined by the proteins either associated with or embedded in the respective membrane. The membrane associated proteins are in this context scaffold proteins such as clathrin, membrane-binding modules such as BAR domains and other cytoskeletal components, which can actively bend regions of lipid membranes [49]. IMPs, in contrast, can influence membranes from their location within the bilayer as every IMP energetically favours a certain membrane curvature for its complementarity to its overall molecular shape. Membrane curvature is furthermore strongly intertwined with the lipid composition of the respective bilayer leaflets. Lipids as such have distinct three-dimensional shapes, which can be broadly categorised into cylindrical, conical and inverted conical, depending on the dimensions of their hydrophobic acyl chains relative to the size of their respective head group. A small head group (e.g., phosphatidic acid) in combination with bulky, e.g., unsaturated acyl chains, yields a conical shape. A bulky head group (e.g., phosphatidylinositol phosphates) in combination with saturated acyl chains or a monoacylglycerol scaffold renders the lipid an inverted cone. Membrane lipids eventually localise in and stabilise preferentially leaflets of a curvature that are complementary to their own shape [49,50].

In their apo state, GPCRs adopt an inverted cone-like shape, which makes them particularly complementary to bilayers that show a positive curvature, as for example in dendritic spines or fillopodia [46]. Interestingly, agonist binding has been found to relax the intrinsic curvature of GPCRs, driving them into a more cylindrical shape [46]. This change in shape is likely explained by the large outward movement of TM6 and the simultaneous constriction of the extracellular half of the receptors observed in the crystal- and cryo-EM structures of ternary GPCR complexes [51]. The change of shape could be an important molecular feature of GPCR desensitisation and internalisation, as it facilitates the localisation of activated GPCRs to negatively curved membranes, such as the ones of early endosomes (see Figure 2) [52]. Several studies on rhodopsin seem further to support the idea that a change in membrane curvature can significantly affect the equilibrium of the different conformational states of GPCRs [53,54].

#### 3.1.3. GPCRs and the Hydrophobic Mismatch

The major characteristic of IMPs is their membrane spanning hydrophobic transmembrane domains (TMDs). TMDs have a defined hydrophobic thickness (d_TMD_) that determines their interaction with the hydrocarbon core of a membrane. A difference in the thickness between d_TMD_ and the hydrocarbon core (d_HC_) is defined as a hydrophobic mismatch (Δd_TMD-HC_). Hydrophobic TM segments are in IMPs further often flanked by so-called anchoring residues [55,56]. These help to position the tips of the TMDs in the interface region of the lipid bilayer. The most prominent anchoring residues are the amphiphilic aromatic amino acids Trp and Tyr [57]. These localise favourably in the bilayer interface region, where they can partition into the hydrocarbon core and at the same time form hydrogen bonds with lipid carbonyls, as well as π-cation interactions with positively charged head group moieties [57,58]. Another class of anchoring residue are the positively charged amino acids Lys and Arg. They are often localised within the hydrophobic TM segments and, with their long and flexible side chains, “snorkel” into the interface region to form stabilising contacts with polar and negatively charged lipid head groups [59,60].

A difference in hydrophobic thickness between TMDs and a membrane, as well as an unfavourable positioning of anchoring residues, are accompanied with a high energetic cost [61]. IMP-membrane systems can minimise that cost in several ways. On the one hand, membrane lipids have a certain elasticity in the z dimension and can thus adapt to a hydrophobic mismatch by either stretching or compressing the length of their acyl chains [62,63]. On the other hand, IMPs can minimise the hydrophobic mismatch by tilting their TMDs relative to the lipid membrane.

The adaption of IMP structure to a given hydrophobic mismatch has been extensively studied for low-complexity model IMPs, such as glycophorin A (GpA). These studies show that TMD tilting in response to a hydrophobic mismatch largely preserves inter-TMD interactions (hydrogen bonds and salt bridges), while the overall structure of the IMP is evidently altered [64]. For GPCRs, studies on the concrete effects of a hydrophobic mismatch are still sparse. However, experimental and computational data suggest that the structure and function of GPCRs can in fact be modulated by the hydrophobic mismatch between a receptor and its lipidic environment [65].

It remains, however, questionable whether these findings are physiologically relevant for GPCRs. While it is known that cellular membranes vary in their hydrophobic thickness [66], their complex lipid composition also allows recruitment processes to take place. Lateral diffusion of lipids and IMPs can lead to phase separation within the plane of the membrane, where lipids and IMPs of similar hydrophobic thickness can cluster together and form nanodomains [67,68]. In the context of GPCRs, this has critical implications for the trafficking and localisation of the receptors [63] and is furthermore likely to regulate the formation of GPCR oligomers [69,70]. The local concentration of certain lipid species in membrane nanodomains may further be an important factor for stoichiometric lipid–GPCR interactions (see Section 3.2) [71].

Molecular dynamic (MD) simulations have revealed structural and kinetic aspects of lipid nanodomains. Recently, all atom MD studies by the groups of Vattulainen and Lyman indicated the presence of substructures within the L_o_ (liquid-ordered) bilayer phase [72,73,74]. In such studies, the slow kinetics of phase separation hampers the ability to observe spontaneous segregation into coexisting L_o_/L_d_ (liquid-disordered) domains. Coarse-grained (CG) models, facilitating longer modelled durations, have proven invaluable in exploring this aspect. Spontaneous formation of L_o_ and L_d_ domains in ternary mixtures of cholesterol, saturated and unsaturated lipids show structural and dynamic properties closely matching experimental data [75]. Further studies have explored the effect of lipid composition on the properties of lipid domains [76,77,78,79].

Several MD simulation studies commonly using a Martini coarse-grained force field (CGFF) were used to explore the oligomerisation tendency of membrane proteins including GPCRs and found evidence for self-assembly. Using Martini coarse-grained molecular dynamic (CGMD) simulations, the oligomerisation tendency of 16 rhodopsins embedded in lipid bilayers of varying thickness was monitored [69]. Maximal dispersion of rhodopsin was found at an intermediate bilayer thickness matching the hydrophobic thickness of the receptor, while its propensity to form inter-protein contacts increased in thinner and thicker bilayers [69]. Mismatch induced oligomerisation can be explained by a minimisation of the energetic cost, which decreases as two proteins experiencing similar hydrophobic mismatch approach one and other [80]. Spontaneous self-assembly depending on the membrane thickness is in agreement with experimental FRET [70] and EM based observations [81]. The latter suggests that a certain level of mismatch can be tolerated, as no significant oligomerisation of bacteriorhodopsin occurs in PC membranes up to 4 Å thicker or 10 Å thinner than the embedded protein [81].

#### 3.1.4. Electrostatic Membrane Potentials and GPCR Function

Biological lipid membranes display complex electric potentials, which can significantly affect the function of GPCRs and other IMPs. A standard electric potential of a cellular membrane can be described in simple terms as the sum of three distinct potentials [30,82]:

Ψ*_tot_*(z) = ∆Ψ(z) + Ψ*_S_*(z)+ Ψ*_I_*(z) (1)

The total electric potential $\Psi_{tot}$ in the z dimension of the membrane can be decomposed into contributions from a transmembrane potential ΔΨ, a surface potential of the lipid head groups $\Psi_{S}$ and an internal electric potential $\Psi_{I}$. The resulting total electric potential profile of a cellular membrane will be similar to the one depicted in Figure 2.

The transmembrane potential ∆Ψ is the electric potential difference between the two aqueous phases separated by the respective membrane. Nearly all biological membranes show such an electric potential difference or are, in other words, polarised. This polarisation can reach considerable voltages of up to −90 mV [83]. This corresponds to energies of approximately 8 kJ mol^−1^ for the transfer of a charge across the membrane (cf. Equation (2)). Given the small dimensions of the membrane, this further amounts to enormous electric field strengths and accordingly very strong electric forces in the lipid bilayer [84].

∆G^0^’ = ∆Ψ × q × F (2)

with ∆G^0^’ being the standard Gibbs free energy for the translocation of a charge q over the membrane, ΔΨ the respective transmembrane potential and F the Faraday constant (≈96,485.3 C mol^−1^).

In the biological context of GPCRs, it is important to appreciate that strong modulations of ∆Ψ (up to ±100 mV) are extensively used by cells for communication and signal propagation [85,86].

Ψ*_s_* is predominantly determined by the head group composition of a lipid bilayer. As biological membranes contain a mixture of neutral, zwitterionic and negatively charged head groups, ∆Ψ usually constitutes a negative electric potential in the interface region of the bilayer [30,87]. This has important implications in the context of in vitro studies of GPCRs. Most membrane mimetics used today lack a negative surface potential. This includes commonly used neutral detergents, as well as zwitterionic membrane lipids used in lipid nanodiscs or nanoparticles. With the ambition to mimic cellular membranes, it is reasonable to include negatively charged head groups in the membrane mimetic [88,89]. Ψ*_I_* comprises the electric potentials of the hydrocarbon core of the lipid membrane. Ψ*_I_*is in this context dominated by the dipole moment of the lipid acyl chains. This dipole moment is caused by the electronegativity of the carbonyl moiety that polarises the entire acyl chain of a lipid [90,91].

These electric potentials have an influence on many membrane properties, such as ion permeability or interactions of the membrane with charged solutes. Most importantly, however, the electric potentials can actively shape the structure, dynamics and function of IMPs. The field of studying these effects on GPCRs is still rather exotic. For other IMPs, including in particular voltage gated ion channels, the significant influence of ∆Ψ is a well understood and appreciated phenomenon [92,93]. An important starting point for considerations of any influence of electric potentials on GPCRs is the discovery that the receptors display defined “gating currents” in electrophysiological terms. On a molecular level this means that charged moieties of the receptors can move in the z dimension in response to changes in ∆Ψ [94]. Over the course of the past two decades, the activation of several GPCRs, including P2YR, all muscarinic acetyl choline receptors (M_1-5_) and the δ-opioid receptor [95,96,97], was shown to be significantly modulated by ∆Ψ.

The concrete molecular mechanisms underlying these activity modulations are still subject to debate. The explanations range today from the movement of charged GPCR residues to allosteric Na^+^ switches that are triggered by changes in ∆Ψ [94,96]. It is important to note that Ψ*_tot_* can, of course, also influence charged ligands and cytoplasmic coupling partners (CCPs) and thereby modulate the apparent activity of GPCRs.

#### 3.1.5. External Factors Governing Membrane Properties

##### The Aqueous Phases Shape Membrane Properties In Vivo and In Vitro

In order to understand and faithfully mimic lipid membranes, it is important to appreciate that their physicochemical properties are critically influenced by the surrounding aqueous compartments. Factors, such as pH, ion concentrations or the presence of small amphiphilic molecules in these compartments have long been known to modulate, e.g., the phase transitions, the permeability or the thickness of lipid bilayers. As outlined in the sections above, these factors have the potential to modulate the structure, dynamics and eventually function of GPCRs. Especially in an in vitro context, buffer conditions should be carefully assessed therefore with regard to the effects they exert on different membrane mimetics.

pH and ion concentrations are factors that are known to strongly affect lipid bilayers. As biological lipid head groups are either neutral, zwitterionic or negatively charged and contain, in most cases, a phosphate moiety, cations such as Na^+^ or Ca^2+^ interact strongly with the interface region of lipid bilayers. As counter ions for negatively charged head group moieties, they generally contribute to the stability of the lipid bilayer. At the same time, cation interactions induce a decrease in the lateral pressure and fluidity of lipid membranes [62]. The coordination of cations by the membrane lipids further leads to an increase in acyl chain order, which causes a thickening of the lipid bilayer and a corresponding loss of elasticity in the z dimension [98,99]. Interestingly, similar effects on the membrane properties are induced by low pH values. It is currently unclear whether these effects are caused by protonation of the lipid head groups [62] or the increased concentration of hydronium ions [100]. It has been shown, however, that a low pH increases the melting temperature and the permeability of lipid membranes and modulates their mechanical properties [101].

Small amphiphilic molecules can partition into the hydrocarbon core of the membrane and can thereby modulate membrane properties. Those molecules include general anaesthetics, such as isoflurane or N_2_O [62], as well as common signalling molecules, such as serotonin [102,103]. Small amphiphilic molecules have been shown to lower, e.g., the melting temperature and to relieve lateral pressure in lipid bilayers [62,104]. These effects can in turn influence GPCRs. For in vitro studies on GPCRs, this consideration has several implications. On the one hand, small amphiphilic molecules can be used to selectively manipulate properties in membrane mimetics. For example, TFE has been added to rule out lateral pressure effects [3]. On the other hand, it implies that GPCR ligands can also have membrane mediated effects, especially at the saturating concentrations of >1 mM often used in experiments [105].

##### Temperature

A key factor shaping lipid bilayer properties is temperature. The temperature effectively determines the energy of a membrane system, which is decisive for many central parameters of a lipid bilayer, ranging from acyl chain order, over lateral fluidity to general phase transitions. In biological membranes lipids are usually in a fluid liquid-disordered (L_d_) or liquid-ordered phase (L_o_), in which they are able to diffuse in the lateral dimension [106]. The transition temperature to a gel-like solid phase is in living cells actively adjusted to remain usually approximately 10–15 °C below the ambient temperature. This is often achieved by an increased incorporation of unsaturated and shorter acyl chains into the membrane [107].

In vitro, however, a maximum of a few lipid species can be used in fixed mixtures. This can result in membrane mimetics with extreme properties. This becomes particularly pronounced with regard to their melting regimes, as commonly used membrane lipids have melting temperatures from, e.g., −18 °C for DOPG to 60 °C for DSPC. This has important implications for the work on GPCRs, since the difference between lipid-melting and ambient temperature can dictate whether the lipid bilayer surrounding the GPCR being studied is in a fluid L_d_/L_o_ or a solid phase [62]. The respective phase state can further affect other membrane properties, such as the thickness or the elasticity of the lipid bilayer [62].

### 3.2. Specific Lipid–GPCR Interactions

With their helical transmembrane core embedded in the phospholipid bilayer, GPCRs are susceptible to the general non-specific properties of the membrane bilayer as described in the previous sections. It is important, however, to understand the impact specific or stoichiometric lipid interactions have on receptor structure and function [108,109,110,111]. Next to, and likely associated with, directly modulating the conformational landscape sampled by a receptor membrane, bound lipids have been suggested also to influence ternary interactions between GPCRs and G proteins [112,113]. One of the earliest indicators for a role of specific lipid interactions was the adverse effects delipidation had on the function of the β_2_ adrenergic receptor [114]. Since then, individual lipid interactions with GPCRs, as well as less specific and general membrane effects have been implicated in disease progression during ageing [115]. These observations emphasize the need to understand the role of lipids for function and organisation of GPCRs under normal and pathological conditions.

Specific lipid interactions could be defined based on the concept of “non-annular” sites of individual lipids in membrane proteins [116,117]. Non-annular lipids are not effectively displaced by competition with annular lipids, hence they are characterised by the lack of accessibility to the bulk lipid pool [118]. The molecular descriptors leading to specific lipid interactions overall are still little understood but are increasingly becoming the subject of experimental and computational studies. Over recent years our understanding of activation mechanism of GPCRs and their dynamic behaviour has been significantly enhanced by all atom MD simulations of these receptors with some focus on β_2_AR as a model protein [119,120,121,122]. MD simulations are now helping to describe, at a molecular level, how lipidic modulators such as cholesterol exert their influence.

#### 3.2.1. Cholesterol

As far as lipid interactions with GPCRs are concerned, studies involving cholesterol are the most abundant. Cholesterol is a modified steroid lipid biosynthesized by all animal cells. As an essential structural component of membranes, it is known to modulate membrane fluidity in relation to changes in the environmental temperature. A short hydroxyl moiety of cholesterol forms the head group of the molecule interacting with the polar phase, whilst planar steroid rings are embedded in the membrane. Cholesterol interactions with proteins have been explored extensively by a range of methods including X-ray crystallography, cryo-EM, NMR spectroscopy and MD simulations [123,124,125]. Lipid rafts present in cell membranes consist of nanoscale domains that impact on structure and function of the proteins embedded within them [126,127]. Changes in the surrounding lipid composition influences GPCRs, which may be triggered by cholesterol promoting the dynamic assembly of such nanoscale membrane domains [74]. Cholesterol modulates the physical properties of membranes by increasing the bilayer thickness and order while slowing the dynamics [79]. As discussed earlier, changes in such general characteristics can influence the dynamic nature of a membrane protein [128]. A variety of experimental studies suggest that cholesterol affects the conformation and function of many GPCRs [108,123,129,130]. However, while cholesterol is a significant component of biological membranes in reference to changes in GPCR function and supramolecular organisation, its mechanism of action is still a matter of debate [131].

Based on the extensive work from the Chattopadhyay lab, Sengupta and co-workers used Martini coarse-grained molecular dynamic (CGMD) simulations to study the interaction of cholesterol with GPCRs and rationalize its effects on their oligomerisation [132]. The interaction pattern of cholesterol with a serotonin receptor (5-HT1A) was characterised for a single receptor molecule embedded into a POPC bilayer containing variable amounts of cholesterol [133]. Overall, cholesterol molecules explored most of the receptor surface during the simulations but spent a significant amount of time bound to specific locations. One of these locations corresponded to one of the consensus cholesterol-binding motifs (CRAC) on transmembrane helix 5 (TM5) [134]. Further work from the Sengupta group simulating the self-assembly behaviour of β_2_AR in a POPC bilayer showed that varying the amount of cholesterol affected the dimerisation interface of the receptors [135]. Without cholesterol, β_2_ARs almost exclusively interact via TM4/5, while with increasing amounts of cholesterol, β_2_AR assembled also via TM1/2 contacts. At 50% cholesterol, β_2_AR switched to an almost exclusive mode of assembly using TM1/2 in a symmetric interface. Occupancy analysis suggested that higher levels of cholesterol resulted in blocking a protein–protein interaction site located at the centre of TM4. This site matched the position of a cholesterol molecule found in a crystal structure [136,137].

**Figure 3:**
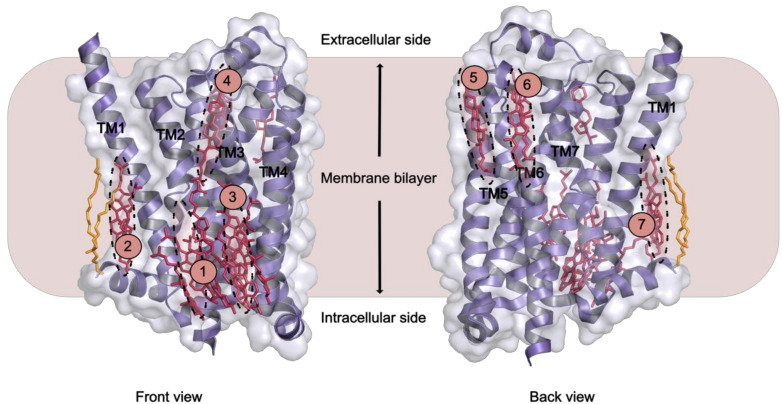
Cartoon overlay of GPCR crystal structures showing different cholesterol binding sites found (PDB accession codes: 2RH1, 3D4S, 4PXZ, 4IB1, 4NTJ, 6WIV, 4EIY, 4DKL, and 4OR2). Structures are aligned with β_1_AR (PDB accession code: 2Y00). The locations of the lipid binding sites are numbered according to Table 1. Cholesterol and cholesteryl hemisuccinate molecules are shown in red (binding sites 1–7), and the palmitoyl lipids are displayed in orange (binding site 2).

Cholesterol is found to be necessary in crystallising β_2_AR [137], and like its soluble analogue cholesteryl hemisuccinate (CHS), it has been shown to improve β_2_AR stability [138]. Crystal structures of β_2_AR showed further that cholesterol binding changed the structural properties of the receptor [136,138]. Other techniques have identified cholesterol binding sites. Two classes of cholesterol binding sites for β_2_AR were found by NMR spectroscopy [139], while MD simulation studies indicated direct interactions between cholesterol and GPCRs, including A_2A_AR and β_2_AR [117,135,140,141]. Concerning GPCRs overall, at least seven distinct cholesterol binding sites have been observed in crystal structures of different receptors [136,137,142,143,144,145,146,147] (Table 1 and Figure 3). MD simulations also revealed seven potential cholesterol binding sites on the surface of β_2_AR. Out of these, three match the ones observed in crystal structures [140]. A different MD study of the same receptor identified three specific high affinity cholesterol interaction sites, two on the intracellular side at the cleft of helices TM5-TM6 and TM1-TM4 with one site on the extracellular side in the TM5-TM6-ECL3-TM7 region [128].The latter two sites agreeing with crystal structures. Further it became obvious that the conformational distribution of β_2_AR drastically altered as the concentration of cholesterol increased to ~10 mol% [136,137,143,148]. The TM1-TM4 cleft showed binding of two cholesterol molecules as predicted for 44% of human class A receptors characterised by the cholesterol consensus motif (CCM) [136]. Mechanistic insight was possibly observed as binding of cholesterol molecules in the TM5-TM6-ECL3-TM7 cleft regulated TM5/TM6 helix movements making it a potential allosteric modulator of receptor activity.

#### 3.2.2. Anionic Lipids

Non-annular lipid binding sites in GPCRs are known to show high selectivity for anionic over zwitterionic lipids. It has been proposed that a change in the type of non-annular lipid alters the packing at protein–protein and/or protein–lipid interfaces modulating the protein activity [149]. The overall strength of specific protein–lipid interactions depends on the net charge of the lipid head groups that can either covalently link to the protein, form specific hydrogen bonds, be involved in ion-mediated salt bridges or take part in electrostatic interactions. The charged protein and lipid components affect membrane partitioning and the translocation thermodynamics [150].

In GPCRs, anionic lipids have been reported to perturb ionic lock formation between the cytoplasmic moieties of TM3 and TM6 and promote receptor activation. MD simulation studies of β_2_AR, showed that a phospholipid from the cytosolic leaflet appeared to insert between receptor helices TM6 and TM7 forming a salt bridge with the guanidino moiety of residue R^3.50^ (Ballesteros–Weinstein numbering) [151] when the receptor was in an active state [152]. The salt bridge acts as a “door stopper” in the activated receptor as it awaits favourable interaction with downstream signalling proteins. Anionic lipids enhance the stability of the receptor active state and increase its lifetime by three-fold, but it is unclear whether this is solely due to the R^3.50^ interaction with lipids.

Increased agonist binding and activation in the presence of phosphatidylglycerol was demonstrated for nanodisc reconstituted β_2_AR [112]. The study went on to show that the increase in activation could also be obtained in the absence of a bilayer while using detergent-solubilized negatively charged lipids, suggesting that the latter were acting as specific allosteric modulators. In agreement with these results, exploring endogenous lipid–receptor interactions for the three class A receptors (β_1_AR, A_2A_AR and NTR1) high-resolution native mass spectrometry (MS) revealed that PIP_2_ stabilises the G protein-bound active state of the receptors and enhances selectivity of coupling to G proteins [31]. The observations were also accompanied by an increase in GTPase activity. Using CGMD simulations, four conserved PIP_2_ binding sites were identified, involving TM1/TM2/TM4, TM3/ICL2/TM4, TM3/TM5 and TM6/TM7.

To characterise the interactions of A_2A_AR with polyanionic phospholipids and glycolipids, CGMD simulations were performed for this receptor in an active and inactive state, or an active state bound to mini-G_s_. All modelled structures were embedded in an in vivo-mimetic membrane [141]. The polyanionic phosphorylated inositol head group of PIP_2_ was found to interact with several basic arginine and lysine residues, with lipid binding concentrating on nine binding sites. While the binding locations for PIP_2_ and GM3 were found to partially overlap, the binding probabilities clearly depended on the activation state of the receptor, in agreement with the earlier studies by Dawaliby et al. [112]. Potential of mean force (PMF) calculations of the free energy landscape for A_2A_AR/PIP_2_ and A_2A_AR/mini-G_s_ interactions suggested a dual role for PIP_2_, promoting both activation of the receptor as well as its association with mini-G_S_. In addition, the study found also that increases in the flexibility of ECL2 as seen in the active state might be related to shortened interaction times of GM3 with ECL2 proximal regions. This would facilitate ligand access to and from the orthosteric binding site, accordingly, influencing the kinetics of ligand binding [141].

#### 3.2.3. Sphingolipids

Sphingolipids constitute 10–20% of total membrane lipids in eukaryotic cells and are known to contribute to lipid segregation into microdomains [153]. Sphingomyelin as the main component is regarded as a reservoir for second messenger molecules such as ceramide and sphingosine-1-phosphate [154]. Specific binding of receptor agonists to the human serotonin 1A receptor was markedly reduced when sphingomyelin in the membrane surrounding the receptor was converted to ceramide by sphingomyelinase with no significant perturbation to the membrane order [155]. This effect on agonist binding was found to be reversible on replenishment of sphingolipids [156]. Prompted by the observed sensitivity of serotonin 1A towards sphingolipids, amino acid sequence analysis led to the proposal of a putative sphingolipid binding domain (SBD) [157], which in parts overlaps with the cholesterol CRAC motif on TM5 [158]. The presence of a similar motif was predicted for the cholecystokinin and the oxytocin receptors, while two SBD motifs were predicted for the secretin receptor [159]. Despite an extensive characterisation of the signalling features the role of sphingolipids in modulating GPCR function remains little understood.

#### 3.2.4. Unsaturated Acyl Chains

As described in Section 3.1, the type of lipid acyl chains influences membrane fluidity, flexibility and lateral pressure. However, they also play a key role in specific interactions with the embedded receptors [160,161]. Unsaturated lipids contain fatty acid side chains with one or more double bonds in their structures, such as palmitoleic acid, oleic acid, myristoleic acid, linoleic acid and arachidonic acid. Eicosapentaenoic acid (EPA), and docosahexaenoic acid (DHA) the most abundant ω-3 fatty acid in human brains [162] are among the very-long-chain polyunsaturated fatty acids obtained from diet or metabolised in small amounts from linoleic acid [161,163,164]. At present, studies on specific effects of unsaturated lipids in the context of GPCR function are still sparse and the effects of unsaturated acyl chains on the receptor conformation may vary for different receptor classes, as many of the lipid-facing residues in the TM regions are little conserved.

For rhodopsin, it was observed using UV–visible absorption spectroscopy that DHA chains facilitated a transition from the light-activated meta-rhodopsin I form to the G protein-binding intermediate meta-rhodopsin II. This transition is characterised by inactive and active conformations of the NPxxY motif on TM7 [165].

A recent NMR study of A_2A_AR embedded in lipid nanodiscs showed that DHA chains increased G protein activation [166]. A_2A_AR bound to the full agonist was found in an equilibrium involving multiple active conformations of TM7. DHA was observed to accelerate conformational exchange and to shift the equilibrium towards a conformation displaying a large clockwise rotation of TM6. The latter was postulated to be preferable for G protein activation.

Combining all atom and CGMD simulations with free energy calculations, it was found that DHA-containing hybrid lipid enhances A_2A_AR partitioning into the Lo phase while in the absence of DHA the receptor partitioning into the Ld phase is energetically favoured [167]. It is believed that this tendency relates to the kinked topology of the A_2A_AR that favours the interaction with DHA and other unsaturated lipids as the first lipid shell surrounding the receptor. Analogous studies with the dopamine D_2_ receptor [168] and a lipidomics-style analysis of the brain-associated glucose transporter (GLUT1) [169] whose function is dependent on poly-unsaturated fatty acids point towards similar partitioning into the Lo phase in the presence of DHA/DHA-containing hybrid lipids. Although GLUT1 is not a GPCR its similar response to DHA-dependent phase partitioning is suspected to relate to a similarly rugged shape of its transmembrane helical domains. In contrast, comparative CGMD simulations performed on ErbB1, VDAC-1 and a GpA dimer did not show any efficient solvation of these proteins [167]. This supports a role for DHA in selective raft partitioning, leading to the modulation of protein–protein or protein–lipid interactions. As DHA favours the solvation of GPCRs it is tempting to speculate that a reduced DHA content would change the signalling properties of receptors through changes in their partitioning.

## 4. Membrane Mimetic Systems for Structural and Functional Studies

### 4.1. Detergents

The first detergents to be used in biochemical studies were naturally occurring bile salts [170] and saponins [171]. However, since the advent of synthetic detergents, a vast array of different surfactants have become available [172]. All detergents follow the same broad architecture, having both hydrophobic tails and hydrophilic head group regions. This amphipathic nature gives rise to their ability, above the critical micelle concentration (CMC), to self-assemble in an aqueous environment [173]. This thermodynamically driven process results in detergent micelles organised with the hydrophilic moieties facing outward to interact with the surrounding water whilst the hydrophobic regions pack together in the centre [174].

The solubilisation of proteins from a lipid membrane environment begins with detergent molecules partitioning into the membrane; this is followed by cooperative binding to the protein leading to micelle formation around the protein separating it from the bulk of the lipid phase [175]. Whilst separation of the target protein from the lipid phase is the main goal, as it allows the protein to be isolated and studied, this can lead to excessive delipidation which can have adverse consequences particularly in the case of GPCRs. As discussed above, the requirement for protein–lipid interactions is becoming increasingly apparent for GPCRs and stripping lipids away from the protein can cause destabilisation and inactivation [176]. This destabilisation can be circumvented by the introduction of thermostabilising mutations. However, these can modify protein dynamics and therefore the constitutive activity of a receptor when compared with its wildtype form [177]. Therefore, choice of a mild detergent that has been shown to allow the retention of key lipid interactions and which only binds to the transmembrane region of a given protein is key for successful studies [178].

The huge range of existing detergents vary greatly in their biophysical properties. However, they can be classified into three major families: ionic detergents such as SDS or deoxycholate carry a net charge and are often strong denaturants; zwitterionic detergents such as LDAO or CHAPS which are not commonly used for GPCRs; non-ionic detergents which are generally milder and have hydrophilic groups most commonly consisting of glucosides or PEG [179]. Due to their milder nature, non-ionic detergents and more specifically alkyl glucosides are most commonly used in the isolation of intact GPCRs for biochemical studies. Generally, there is no one detergent appropriate for all receptors, requiring screening to find a suitable candidate. Indeed, taking a modular approach a recent publication demonstrated that varying tail and head groups can greatly affect the functionality and lipid retention of preparations of NTR1 [180]. Within the family of alkyl glucosides, it seems that varieties with branched alky chains, in particular LMNG, increase the stability of isolated receptors [181,182]. MD simulations comparing LMNG to DDM (a non-branched alkyl glucoside) showed LMNG displayed increased density of alkyl chains covering the hydrophobic region of the receptor and higher levels of hydrogen bonding between detergent head groups. These interactions led to reduced mobility of the receptor [183]. Reduced mobility may, however, be deleterious for studying conformational changes in receptors; investigations by NMR of the β_2_AR in either DDM or LMNG showed the latter to slow ligand induced conformational exchange, attributed to the lower off-rates of this detergent [184].

**Figure 4:**
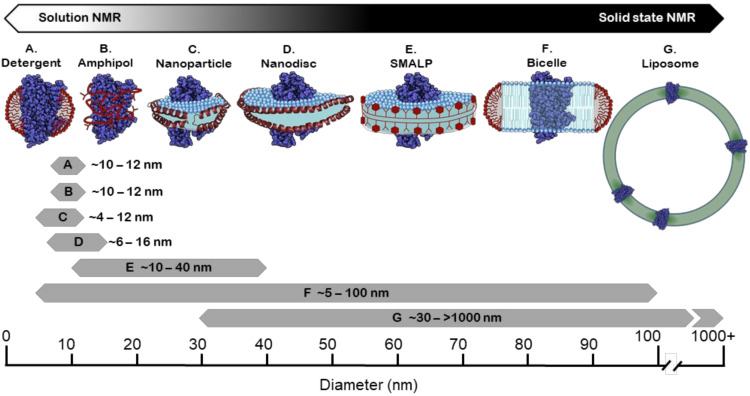
Cartoon representation of frequently used membrane mimetics with an embedded GPCR. With the exception of the Liposome (**G**), the individual mimetic systems are displayed to scale (**A**–**F**). For each system, approximate size ranges are indicated below the individual cartoons. For the non-bilayer-forming mimetics such as detergent micelles (**A**) and amphipols (**B**), size ranges are based on the incorporation of a class A GPCR in LMNG or DDM [216,217], or the A8-35 amphipol [218,219]. The structures for saposin A (**C**), MSPD1 (**D**) and tβ_1_AR (**A**–**G**) were modified from PDB accession codes: 4DDJ, 6XBD, 4BVN, respectively.

The majority of studies on isolated membrane proteins are still carried out in a detergent system; GPCR research is no exception (Table 2) where well-established protocols have greatly simplified membrane extraction. The vast majority of GPCR structures deposited in the PDB have been obtained for receptors in detergent micelles [185]. Further, the proven ability to make homogeneous particles with sizes (Figure 4) that relate to sufficiently rapid rotational tumbling means detergents have been a favoured system for solution NMR studies. This has facilitated the extensive exploration of the conformational exchange dynamics of isolated receptors and revealed GPCRs as populating multiple equilibria between different states (Figure 1B). Such studies have been key to understanding phenomena such as allosteric coupling, ligand efficacy, partial agonism and signalling bias [186,187]. Interactions with specific lipid bilayer components can also be probed by forming mixed micelles of detergent and lipid at a controlled ratio. This has been used to demonstrate that the cholesterol derivative CHS stabilised the A_2A_AR receptor [188]. Indeed, CHS has become a common additive to detergent solubilised receptors in many NMR studies (Table 2), enabling longer experiment times [143,189,190]. In other studies mild detergents such as LMNG and DDM that reduce the delipidation of GPCRs allowed interactions of receptors with endogenous lipids to be elucidated. For example, mass spectrometry combined with activation assays demonstrated the specific binding of PIP_2_ and its relevance in purified class A receptors coupling to Gα [31].

Ultimately, however, a detergent micelle offers a poor mimic of the lipid bilayer. Detergent molecules have hydrophobic tails that are short and highly mobile as compared to the alkyl tails of lipids, this means the isolated protein can dictate the thickness and shape of the hydrophobic phase, allowing non-physiological conformations to be adopted [215]. The hydrophobic packing in micelles is weak compared to a membrane allowing increased water penetration which has been suggested to destabilise receptors [216]. Further, the relatively large ratio of hydrophilic to hydrophobic moieties seen in detergents results in much broader dielectric gradients than observed in a membrane [215]. Furthermore, detergents can destabilise isolated globular proteins reducing the likelihood for GPCRs to be studied in the presence of their native binding partners. Although detergents will likely remain a vital tool for GPCR research, the use of mimetics more faithful to native conditions will no doubt prove necessary for probing receptor structure and function, whilst facilitating the move away from the need for thermostabilising mutations.

### 4.2. Amphipols

Amphipathic polymers, or amphipols, were developed in the 1990s by Popot and co-workers [220] as an alternative to detergents. There is a growing family of amphipols but all have a hydrophilic backbone, commonly polyacrylate, with various hydrophobic side chains [221]. There are very few examples of proteins being directly extracted from native membranes by amphipols and general procedures require detergent solubilised protein first, the detergent is then exchanged for the chosen amphipol [222]. In fact, amphipols are able to refold protein that has been solubilised by denaturing ionic detergents resulting in stable protein. A number of human GPCRs treated in this way have been shown to maintain ligand binding competency, for example the growth hormone secretagogue receptor (GHSR), CB1 cannabinoid and 5-HT4 serotonin receptors [223,224].

Amphipols have very high affinity for the hydrophobic transmembrane regions of proteins and form a well-defined structure around the target protein with bound lipids possibly left intact; however, they do not feature a bulk lipid phase [225]. The relatively high affinity for hydrophobic regions of protein means that only low concentrations of amphipols are required with very little left free in solution. It is thought that the lack of free amphipols in samples prevents them from, unlike detergent micelles, acting as a hydrophobic sink for non-covalently bound molecules such as lipids. Due to the lack of exchange between soluble particles, amphipols are significantly less destabilising than detergents [226]. However, in terms of physiological representation of a membrane bilayer, amphipols have little advantage above detergents [222], lacking for example membrane curvature or the electrostatic effects of phospholipids. Further, the lack of exchange of lipids, whilst it may be stabilising, complicates the study of specific effects of exogenous lipids. Furthermore, it was observed in structures of the TRPV2 ion channel that amphipols constricted the overall structure preventing conformational changes in the transmembrane domain [227]. This suggests that the use of this membrane mimetic may interfere in the study of membrane protein dynamics.

As alluded to, many reports suggest that amphipols are highly stabilising for membrane proteins including GPCRs [228]. Coupled with a modest size increase, this makes them a promising tool for solution-state NMR (Figure 4) [229]. Detailed structural characterisation of the lipidic ligand leukotriene B4 (LTB4) by NMR showed it to adopt a constrained hook shaped conformation when bound to the BLT2 receptor. This was facilitated by the isolation of receptor in partially deuterated amphipols [230]. Recently, ligand selectivity was examined by comparing the binding of LTB4 and agonist 12-HHT to the same receptor in perdeuterated amphipols [231]. While structural information on various 7TM receptors, such as bacteriorhodopsin and melanocortin-2 and -4, has been reported in solution [232,233], it is not known whether these investigations using amphipols have progressed beyond a proof of principle. Amphipols have also proved useful in other biophysical studies of GPCRs. V2 vasopressin receptor isolated into amphipols was shown to activate G protein, and conformational changes consistent with biased agonism were observed using TR-FRET and tryptophan fluorescence [219]. A key positive aspect of amphipols is that they are open to chemical modification; affinity tags can be introduced allowing for receptor–amphipol complexes to be immobilised, facilitating ligand-screening techniques as trailed with the ghrelin receptor [234].

### 4.3. Bicelles

Bicelles are discoidal structures composed of long-chain phospholipids, most commonly DMPC, and a detergent, typically CHAPSO or DHPC (a detergent-like short-chain phospholipid). When prepared, provided that the phospholipid to detergent ratio (*q* value) is high enough, the components arrange such that a planar phospholipid bilayer surrounded by a rim of detergent molecules is formed [235]. Protein reconstitution can be carried out either by adding protein solubilised in detergent to phospholipid so that the desired *q* value is reached, or by solubilising pre-reconstituted proteoliposomes [236].

For a given lipid and detergent pair, the *q* value dictates the eventual size (Figure 4) and morphology of the bicelle. This means that by altering *q*, the suitability of this system can be adjusted for a range of biophysical techniques. For example, DMPC-DHPC bicelles prepared with *q* values > 2.3 will align with a magnetic field and can be used in oriented-sample solid-state NMR in order to establish ^15^N-labelled amide bond orientations and the TM helix angle of the incorporated proteins relative to the bilayer normal [237,238], whilst *q* values ≤ 0.7 allow bicelles to be sufficiently small for solution-state NMR to be carried out [239].

However, the *q* value also dictates how well the bicelle might represent a true bilayer environment. Looking at backbone amide signals of the Fas TM domain in conjunction with solvent paramagnetic relaxation enhancement (PRE), it was found that *q* values < 0.5 caused transmembrane residues to be more solvent exposed, suggesting significant hydrophobic mismatch [240]. Other researchers showed that, in terms of morphology and phase behaviour, bicelles with *q* values < 1 deviated from true bilayer systems, whilst they essentially represented mixed micelles with no spatial separation between surfactant and phospholipid at *q* < 0.5 [241]. Contrastingly, ^31^P NMR has been employed to show that DMPC and DHPC head groups were well segregated even at *q* values below 0.5 [242]. Also, others produced data suggesting that low *q* bicelles are discoidal and as such represent a bilayer patch [239] and that this behaviour was consistent over a broad range of phospholipid concentrations (50–300 mM) [243]. Therefore, it remains unclear whether a lipid:detergent “sweet spot” can be achieved when using bicelles as membrane mimetics in solution NMR. Problems can also arise as depending on the CMC value of the detergent employed to form bicelles, the concentration of monomeric detergent can be substantial in solution and interfere with the study of globular proteins such as GPCR binding partners or the extramembranous regions of a receptor.

However, bicelles do have the advantage of having a highly tuneable composition. This can allow solution and solid-state NMR to be used in a complementary way with only minimal changes to sample preparation. This is demonstrated for the Y2 receptor which was refolded from SDS purified samples into bicelles of low or high *q* [244]. Furthermore, whilst the canonical bicelle system is formed of DHPC and DMPC, other mixes can be used. Researchers have made bicelle-like preparations using DDM and CHS that stabilised the ORL-1 receptor [245]. The effect of different lipid acyl tails (DMPC vs. POPC) on GHS receptor dynamics was examined in bicelles by MAS solid-state NMR [246]. Further, bicelles rich in sphingolipids and cholesterol were recently shown to form a true bilayer even at low *q* (>0.25) [247], indicating that the use of bicelles in solution-state NMR-based GPCR studies may just be a matter of further screening for optimal bicelle components. Even if doubts of bilayer “likeness” might be an issue for some preparations, bicelles do appear to perform better when examining GPCRs by hydrogen deuterium exchange (HDX). Better sequence coverage and more peptide redundancy were observed for β_2_AR, μOR and PAR1 in bicelles compared to DDM detergent micelles, leading to higher-quality HDX data [248].

### 4.4. Liposomes

Liposomes are vesicular structures consisting of a lipid bilayer enclosing an aqueous space. They have been a long-standing system for the study of membrane proteins and membrane dynamics [249,250]. Structures with multiple concentric bilayers will form spontaneously upon the suspension of a dried phospholipid film in an aqueous buffer; these are termed multilamellar vesicles (MLVs). However, MLVs are generally of little use for biophysical studies, as they have an onion-like structure with high heterogeneity [251]. Therefore, typically, preparations of MLVs are treated, for example, by extrusion or sonication in order to yield uni-lamellar vesicles (ULVs) of a more uniform size [252]. The ultimate size distribution of the ULVs depends on the method of formation and the phospholipid composition used and can range from small uni-lamellar vesicles (SUVs, 20–100 nm) to large uni-lamellar vesicles (LUVs, 100–1000 nm) and giant uni-lamellar vesicles (GUVs, >1000 nm) [253]. Even within classical preparations of ULVs, there can be heterogeneity and efforts have been made to generate liposomes of highly controlled monodisperse sizes. To this end, researchers developed a DNA templating technique allowing the formation of liposomes of a desired size with nanometre precision in 2016 [254]. The reconstitution of proteins within the liposome bilayer typically proceeds by partially solubilising pre-formed ULVs with detergent, adding detergent solubilised protein and removing the detergent with bio-beads or dialysis to reform an intact bilayer containing the embedded protein. This has facilitated the study of a huge array of membrane proteins, including GPCRs in a controlled membrane environment [255,256,257,258].

Compared with all the membrane mimetic systems explored in this review liposomes offer several unique advantages. First, the large size of liposomes allows for multi component systems (proteins, ligands and substrates) to be reconstituted [259]. Furthermore, the aqueous lumen can encapsulate soluble components allowing entire GPCR signalling pathways to be reconstituted, for example the M1 muscarinic receptor along with G_q/11_βγ and phospholipase C in liposomes were induced to produce inositol triphosphate upon exposure of carbachol [260]. Secondly, due to the compartmentalisation of an aqueous phase by a hydrophobic bilayer, as with a native cell, liposomes are able to sustain a membrane potential [261] giving the opportunity to study the effect of ∆Ψ on receptor function. However, dictating the orientation of the reconstituted GPCR to obtain meaningful results can be a complicating factor. Finally, in contrast to the other mimetics discussed here that approximate a bilayer structure, liposomes are unconstrained by a solubilising scaffold structure and represent a continuous membrane. This continuous nature allows for native lateral pressure and diffusion behaviour of phospholipids and proteins to be studied, and it also allows for the membrane components to induce membrane curvature [262]. However, curvature can deviate from that experienced in a native membrane when smaller more constrained SUVs are used [263].

Generally for liposomes, there are no limitations with regard to the complexity of the lipid composition that can be used, enabling the assembly of extended bilayers based on cellular lipid extracts. This also gives the opportunity to define the lipid composition, allowing the study of specific lipid interactions. For example, the phenomenon of receptor dimerisation was studied using FRET on fluorescently labelled neurotensin receptors in liposomes to show that the presence of cholesterol and PE-lipids stimulated dimer formation [264].

Unfortunately the large size of liposomes even when using SUVs generally precludes their use for solution NMR studies (Figure 4) aiming at a characterisation directly on the intact receptor [237]. However, waterLOGSY-based NMR spectroscopy has been successfully applied to study the membrane association of the truncated C terminus of the B-type CGRP receptor [265]. With the rapid advancement of solid-state NMR techniques, the investigation of GPCRs embedded in liposomes has progressed as shown for the investigation of CXCR1 by a combination of magic angle spinning and oriented-sample NMR [266]. Further, solid-state NMR has been employed to study ligand binding where challenges with studying high-affinity receptor NTR1 were tackled [267].

### 4.5. Membrane Scaffold Protein (MSP) Nanodiscs

The family of apolipoproteins (Apo) is responsible for facilitating the transport of lipids in the bloodstream. Specifically, Apo-A1 is a key structural component of high density lipoprotein (HDL) that forms a scaffold surrounding phospholipids and cholesterol [268]. In 2002, Sligar and co-workers modified the *APOA1* gene, removing the globular domain, and demonstrated that it could assemble around phospholipids to form a homogeneous population of particles with a discoidal structure. They termed the modified protein an MSP and its complex with lipids a nanodisc [269]. Since then, a family of MSPs has been developed by further modifying the number of helical repeats that ultimately encircle the nanodisc, each variant forming discs of a different diameter (~6–16 nm Figure 4) [270,271,272]. Nanodiscs are formed by mixing detergent-solubilized phospholipids and the MSP together followed by subsequently removing the detergent typically with bio-beads. In the presence of a detergent-solubilized protein, the latter is also incorporated into the disc, where it is surrounded by lipid molecules [273].

Structural studies show that nanodiscs contain a patch of lipid bilayer encapsulated with two MSPs running antiparallel and stabilized by a zipper-like arrangement of salt bridges between the proteins [274]. For an empty disk, the number of phospholipids in each particle range from ~20 to 400 depending on the MSP construct and the type of phospholipid used, with unsaturated alkyl chains packing less tightly into the disc [270,271,275]. Shorter MSPs generating smaller discs may be advantageous for solution NMR, as demonstrated by Wagner and co-workers, who found that in studies of OmpX, nanodiscs of 8.4 nm provided a suitable compromise between stability and NMR data quality [270]. However, it should be considered that with reduced disc sizes, likelihood of interactions between the MSP and phospholipid molecules increases, reducing the number of free lipids. Smaller discs also increase the risk of spurious interactions between the extramembranous regions of the embedded protein and the scaffold protein [273]. Commonly used variants in solution-state NMR studies of GPCRs are MSP1D1 and MSP1D1E3, which give disc sizes of ~9.6 and ~12 nm, respectively, requiring deuteration of the receptor for sufficient spectral quality [166,196].

The cryo-EM structure of the SecYEG complex in a nanodisc is consistent with a true lipid bilayer with strong density observed for the phospholipid head groups at the surfaces [276], suggesting that the electrostatic profile of the bilayer is maintained. Biophysical studies showed that, whilst the DPPC and DMPC nanodiscs did display classical crystalline to liquid phase transition, this occurred at a higher temperature; a result attributed to the MSP itself causing increased lateral pressure compared with a continuous bilayer [277]. Overall, the nanodisc system represents a vast improvement over detergents in its ability to emulate a membrane environment. There are still subtle differences that need to be taken into account however. For example, the bilayer patch in standard-size nanodiscs appears too small for a membrane component to impose curvature or to study the effects of bilayer asymmetry. However, technologies such as DNA-corralled nanodiscs (DCNDs) facilitate the formation of much larger structures (≤90 nm diameter) and may allow the study of proteins in the context of these phenomena [278].

The use of MSPs in both functional and structural studies of GPCRs is becoming increasingly common (Table 2), offering insights beyond those carried out in detergent micelles. Using NMR, Kofuku et al. compared the dynamics of the β_2_AR in MSPs and DDM micelles, finding the former to cause the ligand-bound β_2_AR to populate an active conformation to a greater extent and exhibit slower rates of conformational exchange [196]. The mimetic system allows the lipid content surrounding the protein to be defined and as such probed for specific effects. Nanodiscs were used in conjunction with microscale thermophoresis to indicate that more “native-like” PE-rich membranes increase the affinity of NTR1 to Gα_i_ [279]. MSPs have also facilitated studies looking at the relevance of specific varieties of alkyl tails on receptor activity. It has been demonstrated that the presence of DHA within the nanodisc stimulated G protein activation significantly, possibly by stabilising receptor conformations that couple more favourably to Gα, as observed by NMR [166].

The number of MSP variants continues to expand introducing more advantageous properties. Covalently circularised nanodiscs (cNDs) have been used to study a thermostabilised NTR1 by NMR, demonstrating higher stability and homogeneity than in detergent [203]. cNDs have also been used for the cryo-EM structural determination of NTR1 and Gα_i_. Interestingly, a higher degree of contact was observed between the two proteins relative to detergent based structures. This was attributed to the lateral membrane pressure restricting helical movement such that the receptor fits more tightly to the G protein [280]. However, non-physiological levels of lateral pressure may be forcing this interaction. Using a protein scaffold-based membrane mimetic such as MSP also has the advantage of allowing the easy introduction of affinity tags to the complex; this can assist in purification and biophysical experiments. Strep-tagged MSP was used to observe receptor dynamics through fluorescence changes of individual labelled receptors bound to a streptavidin-coated surface [4].

### 4.6. Styrene Maleic Acid (SMA) Copolymer and Copolymer Variants

All the membrane mimetic systems discussed thus far require a target protein to first be solubilised in detergent before reconstitution into the system of choice. For GPCRs, this initial detergent step frequently defines the minimal level of stability required for a receptor to be studied and exposes the protein to the potentially disruptive effects of stripping innate phospholipids away (discussed above). However, styrene maleic acid copolymers (SMA) offer a way to circumvent this, having the effect of excising the target protein within a raft of the lipid membrane it originated from, resulting in SMA lipid particles (SMALPs) [281,282]. SMA consists of alternating monomers of hydrophobic styrene and hydrophilic maleic acid that can be incorporated into the polymer at varying ratios. In solution with a lipid bilayer, the styrene moieties insert into the bilayer interacting with the acyl tails. As increasing SMA molecules bind to the membrane, this causes disruption, seen as pore formation [283] and ultimately fragmentation [284]. The hydrophilic maleic acid moieties reside on the outside of the resulting membrane disc, like a protective cage, rendering the whole structure soluble [281]. As the SMA polymer acts to solubilise the entire ~10–40 nm discs (Figure 4) of membrane, any proteins within the membranes are solubilised also meaning the protein never leaves its native local environment [285]. SMA is only the most commonly used polymer of a growing family, as some effort has been placed in altering the hydrophobic and hydrophilic moieties to give more favourable physical characteristics such as improved pH and salt tolerance, and tuneable disc size [286,287].

Previous work has shown that the SMALPs represent a mimetic system that remains faithful to the native environment in a number of ways. Small-angle neutron scattering experiments on DMPC-SMALPs show them to be ~4.6 nm thick, and this is in close alignment with the bilayer thickness of DMPC liposomes [288], suggesting that a full bilayer is maintained. Further, phase transition temperatures for SMALPs are remarkably close to those of lipids in continuous membrane systems. However, the transition is significantly broadened over a larger temperature range, suggesting a drop in cooperativity amongst the lipids caused by interactions with the polymer [288]. When studying endogenously expressed protein, the physiological accuracy of SMALPs is improved over other systems, as the solubilised protein is isolated with a sample of mixed lipid species, representative of the membrane into which the protein was expressed. Coupled with lipidomics, this has helped to delineate the different lipid environments within a cell and their relevance for protein function [289,290].

Structural studies where the protein was solubilised with SMA are few at the time of writing and it remains to be proven how useful the system will be. Extant publications do suggest that interactions with bound lipids remain intact. A cryo-EM structure of the multidrug transporter AcrB exhibited a patch of 24 highly ordered lipids within the AcrB trimer whilst the lipids in the surrounding annulus were relatively disordered [291]. Further, the structure of bacterial complex III cytochrome oxidase supercomplex displayed a number of surface lipids that could be resolved [292]. Of course, it should be noted that SMALPs offer a less controlled system compared to others presented here, with little influence over lipids and membrane components that get extracted with the protein.

SMALPs too exhibit increased thermostability [287], making them particularly attractive to GPCR research as this may ultimately reduce the need for thermostabilising mutations [282]. The only X-ray crystallography structure facilitated by the use of SMALPs was obtained at 2.2 Å for bacteriorhodopsin using lipidic cubic phase [293]. More commonly, GPCR-SMALPs have been applied to biophysical techniques. Intramolecular FRET has been used to monitor conformational changes in SMALP solubilised ghrelin receptors upon ligand binding. Logez et al. critically demonstrated G protein activation by GPCR-SMALPs by means of a GTPγS assay [294]. The system has also been used in the study of receptor dynamics, where the A_2A_AR receptor in SMALPs was measured by fluorescently tagging the base of TM6. As the environment of the tag was modulated by agonist or antagonist binding, conformational changes could be detected. This was used to differentiate between agonists of varying efficacy [295].

Unfortunately, the large size of SMALPs coupled to relatively heterogeneous preparations makes them an unappealing system for solution NMR and as yet no GPCRs have been studied in this manner. However, larger SMALPs (>30 nm) have been shown to align in a magnetic field making them suited to oriented-sample solid-state NMR. As such, structural information has been gained on a small but growing number of membrane proteins isolated in the native-like environment of SMALPs, including the CXCR1 receptor [285,296].

### 4.7. Saposin Nanoparticles

Saposin A (SapA) is a sphingolipid-activating protein, one of four non-enzymatic small proteins (A–D) required for the processing of sphingolipids by hydrolases within lysosomes [297]. All saposins are able to bind certain lipids as part of their biological function and in 2012 SapA was shown to form a disc-like complex with various lipids [298]. SapA monomers are 9 kDa proteins, consisting of four α-helices that, if in the apo state, will form a closed conformation at neutral pH. At acidic pH, as experienced in the lysosome, SapA will open like a jack knife exposing a hydrophobic inner face that can accommodate phospholipids. In complex with LDAO, two SapAs were observed to arrange head to tail, forming a scaffold around a well-ordered bilayer-like core of the zwitterionic detergent [298].

The characteristics of SapA showed clear potential to form the scaffold component of a membrane mimetic system, stabilising a phospholipid bilayer patch. Exploring this further, Frauenfeld et al. crucially demonstrated that SapA opening and phospholipid uptake could be facilitated at neutral pH in the presence of detergent. In the same work, it was shown that a membrane protein could be incorporated into the centre of the nanoparticle in analogous fashion to MSP nanodiscs, i.e., by incubating SapA, detergent-solubilised lipid and protein together and gradually removing the detergent using bio-beads to form a discrete bilayer system [299]. Since then, SapA nanoparticles have been shown to be a tuneable system, where adjusting the phospholipid to SapA ratio or pH can modify the particle size [300,301]. For occupied nanoparticles, it appears, however, that the properties of the embedded protein are primarily dictating the final size of the assembly, with a minimal number of SapA molecules recruited to encircle the membrane protein [301,302].

MD simulations suggest that the 16:0–18:1 chain lipid POPC can form a true bilayer patch with both leaflets intact similar to the LDAO associated structure [298]. Furthermore, in the recently published structure of the bacterial AcrB efflux pump in SapA nanoparticles, a full bilayer can be modelled [303]. Accordingly, as expected of an improved bilayer membrane, mimetic hydrophobic mismatch would be avoided and the electrostatic effect of the bilayer on the protein, in principle, maintained.

The SapA system seems to show a tendency to minimise the circumference of the bilayer surrounding the reconstituted protein [301]. In the structure obtained of the mitochondrial calcium uniporter (MCU), whilst the lipids were unresolved, the six encircling SapA units could be seen to tilt at a 45° angle to fully span the transmembrane domain [304]. While it is currently not clear whether the observed tilt of the scaffold proteins is a more general feature that also applies to other saposin complexes, the 45° tilt in this particular case effectively reduces the overall size of the nanoparticle. This size minimisation of a bilayer system makes the SapA nanoparticles highly attractive for solution NMR (Figure 4). However, this also means that there is only a small lipid phase present and it is possible that in some cases only a lipid annulus exists.

SapA nanodiscs have great potential in the study of GPCRs. Of all the saposins, SapA has been shown to be the most promiscuous in the phospholipid species it will interact with [302]. This will allow the system to be used to examine effects from a wide range of lipids on GPCR function. Already the turkey β_1_AR receptor in SapA nanoparticles showed agonist-dependent activation and ternary complex formation as revealed by NMR, paving the way for more in depth studies to be carried out in a lipid bilayer environment [301]. Recently, using the CK receptor as an example, it was shown that GPCRs can be extracted directly from biological membranes using SapA. While this could circumvent the need for thermostabilising mutations, the high amount of SapA required to solubilise the proteins so far has led to low yields, limiting the use on a preparative scale [305].

## 5. Conclusions and Outlook

Due to the innate difficulties of working with integral membrane proteins, the path to better understanding their structure and function has been a difficult one. Of course, GPCRs are no exception. However, an increasing appreciation for the relevance of lipid bilayer effects coupled to ingenious developments to mimic and study those effects are driving progress in GPCR research. 

Where once detergents and liposomes, as the only controlled systems available, stood at polar opposites in terms of molecular size, tractability and physiological accuracy, there is now a family of different mimetic systems each being well suited to a range of biophysical techniques. These new mimetic systems can be selected also to improve the stability of a GPCR or, crucially, in the case of true bilayer-forming systems such as nanodiscs, to shed light on the specific effects of lipid subtypes. The latter have been put to particular use in NMR-based GPCR studies, where they have started to reveal an ever-richer conformational energy landscape.

Progress in understanding lipid bilayer effects on GPCR dynamics and structure is set to continue. More diverse structural information is being gained on the one hand from static cryo-EM and X-ray studies and on the other from solution-based fluorescence and NMR techniques. Further, indeed, in the case of GPCRs, computational approaches are well placed to link this information together into a cohesive picture. Furthermore, approaches to study membrane proteins in a more native environment continue to develop, as shown for example by a recent demonstration of cryo-EM in a continuous bilayer [306].

## Figures and Tables

**Table 1:** Lipid binding sites in GPCR crystal structures for cholesterol (C), cholesteryl hemisuccinate (CHS) and palmitate (P). The number of bound lipids and the binding interfaces are indicated (IC, intracellular cleft; EC, extracellular cleft; ECL, extracellular loop; ICL, intracellular loop). The locations of the individual binding sites 1–7 are shown in Figure 3.

Site	GPCR	PDB IDs	Number of Bound Lipids	Binding Interface	Crystal Contacts (C) or Dimer Interface (D)
1	β_2_AR	2RH1, 3D4S	2 × C	IC; helices I–IV	C
1	β_1_AR	2Y00	1 × CHS	IC; helices II–IV, ICL1	C
1	P2Y_12_	4PXZ	1 × C	IC; helices II–IV, ICL1	C
2	β_2_AR	2RH1	1 × P	IC; helices I, VIII, palmitate	D
2	5-HT2B	4IB4	1 × P	IC; helices I, VIII, palmitate	D
3	β_1_AR	2Y00	1 × CHS	IC; helices III–V	C
3	P2Y_12_ GABA_B_	4NTJ 6WIV	1 × C 2 × C	IC; helices III–V IC; helices III–V	C D
4	A_2A_AR	4EIY	1 × C	EC; helices V–VI	C
4	β_1_AR	2Y00	1 × CHS	EC; helix V	C
5	A_2A_AR µ-OR	4EIY 4DKL	11 × C	EC; helices VI–VII, ECL3 EC; helices VI–VII, ECL3	
6	P2Y_12_	4NTJ	1 × C	EC; helices VII, I	
7	A_2A_AR	4EIY	1 × C	EC; helices II–III, ECL1	C
7	mGlu1	4OR2	3 × C	EC; N-term, helices I–III, ECL1	D

**Table 2:** Studies of GPCR conformational dynamics using solution-state NMR in conjunction with various membrane mimetics.

Receptor	Expression System	Membrane Mimetic	Labelling	NMR Experiment	Reference
β_2_AR	*Sf*9	DDM	^13^CH_3_-Lys (reductive methylation)	STD-filtered ^1^H,^13^C HMQC; ^1^H,^13^C HSQC	[191]
β_2_AR	*Sf*9	DDM/CHS	^19^F-TET	^19^F; 1D	[143]
β_2_AR	expressSF+	DDM	^13^CH_3_-Met or α,β,β-^2^H_3_,^13^CH_3_-Met	^1^H,^13^C-HMQC	[192]
β_2_AR	*Sf*9	DDM/CHS or LMNG	^19^F-BTFA	^19^F; 1D, T_1_, T_2_	[184]
β_2_AR	*Sf*9	LMNG	^19^F-BTFA	^19^F; 1D, T_1_, T_2_	[193]
β_2_AR	*Sf*9	DDM	^13^CH_3_-Met	^1^H,^13^C HSQC	[194]
β_2_AR	*Sf*9	DDM/CHS	^19^F-TET	^19^F; 1D, 2D EXSY	[195]
β_2_AR	expressSF+	POPC/POPG nanodiscs	b_2_AR [^2^H-9AA, abg ^2^H,^13^CH_3_-Met]	^1^H,^13^C-HMQC; ^1^H,^15^N	[196]
β_2_AR	*Sf*9	LMNG	^19^F-BTFA	^19^F; 1D, CPMG, STD	[197]
mOR	*Sf*9	LMNG/CHS	^2^H-8AA, ab-^2^H-^13^CH_3_-Met	^1^H,^13^C-HMQC	[198]
mOR	*Sf*9	LMNG/CHS	^13^CH_3_-Lys (reductive methylation)	^1^H,^13^C-HMQC	[189]
b_1_AR	*Sf*9	DDM	u-^2^H,^15^N	^1^H,^15^N TROSY	[199]
b_1_AR	High five	DM	^15^N-Val	^1^H,^15^N HSQC	[200]
A_2A_AR	*P. pastoris*	LMNG	^19^F-BTFMA	^19^F; 1D, STD	[201]
BLT2	E. coli	DMPC/CHS nanodiscs	U-^2^H, ^13^CH_3_-δ1-Ile, ^13^CH_3_-ϵ-Met	^1^H,^13^C-HMQC	[123]
b_1_AR	*Sf*9 or *Sf*21	LMNG	^13^CH_3_-Met	^1^H,^13^C-HMQC	[6]
A_2A_AR	*P. pastoris*	DDM	^13^CH_3_ Ile d1/^2^H	^1^H,^13^C HMQC, 3Q-relaxation	[202]
NTR1	*E. coli*	DMPC/DMPC nanodiscs	U-^2^H, ^13^CH_3_-δ1-Ile,^13^CH_3_-ϵ-Met]	^1^H,^13^C-HMQC	[203]
A_2A_AR	*P. pastoris*	LMNG/CHS	U-^15^N, 70% ^2^H	^1^H,^15^N TROSY	[204]
A_2A_AR	*P. pastoris*	LMNG/CHS	U-^15^N, 70% ^2^H	^1^H,^15^N TROSY	[190]
CCR5	*Sf*9	DDM	U-^2^H,^15^N	^1^H,^15^N TROSY	[205]
NTR1	*E. coli*	DDM	^13^CH_3_-Met	^1^H,^13^C-HMQC	[206]
β_2_AR	b_2_AR: expressSF +C-terminal tail: *E. coli*	POPC/POPG nanodiscs	b_2_AR [^2^H-9AA, abg^2^H,^13^CH_3_-Met] C-tail: U-[^2^H, ^13^C, ^15^N] or ^13^CH_3_ Thr g2 and Ile d1	^1^H,^13^C-HMQC; ^1^H,^15^N HSQC; cross-saturation	[207]
β_2_AR	expressSF+	β-DDM or POPC/POPG nanodiscs	α-2H,^13^CH_3_-Ala; αβγ-2H,^13^CH_3_-Met; ^13^C-Ile; ^13^C-Leu; ^13^C-Thr; <80% ^2^H	^1^H,^13^C HSQC; ^1^H, ^13^C TROSY	[208]
A_2A_AR	*P. pastoris*	LMNG	^19^F-BTFMA; metal ions	^19^F, ^23^Na^+^, ^25^Mg^+^; 1D, CPMG	[209]
A_2A_AR	*Sf*9	DDM/CHS	^19^F-TET (in membrane labelling)	^19^F; 1D, 2D EXSY	[210]
M2R	*Sf*9	LMNG/CHS	^ 13 ^ CH_3_-ε-Met	^1^H,^13^C HSQC	[211]
β_2_AR	*Sf*9	LMNG/CHS or POPC/POPG nanodiscs	^19^F-BTFMA	^19^F; 1D	[212]
β_1_AR	*Sf*9	DM	^15^N-Val	^1^H,^15^N TROSY	[213]
β_2_AR	*Sf*9	LMNG	[2,3,3-^2^ H, ^15^N]-Leu, MSTL	^1^H,^15^N TROSY, PRE	[3]
β_1_AR	*Sf*9	LMNG	^19^F-TET	^19^F; 1D, CPMG, STD	[7]
A_2_AR	*P. pastoris*	nanodiscs (POPC/POPG and/or SAPC or SDPC)	α,β,β-^2^H,^13^CH_3_] Met, u-^2^H	^1^H,^13^C-HMQC; ^1^H 1D; ^31^P 1D; solution PRE	[166]
α1AR	*E. coli*	DDM/CHS	^13^CH_3_-ϵ-Met	^1^H,^13^C-HMQC	[214]
